# Reduction of the HIV-1 reservoir in resting CD4^+ ^T-lymphocytes by high dosage intravenous immunoglobulin treatment: a proof-of-concept study

**DOI:** 10.1186/1742-6405-6-15

**Published:** 2009-07-01

**Authors:** Annica Lindkvist, Arvid Edén, Melissa M Norström, Veronica D Gonzalez, Staffan Nilsson, Bo Svennerholm, Annika C Karlsson, Johan K Sandberg, Anders Sönnerborg, Magnus Gisslén

**Affiliations:** 1 Department of Laboratory Medicine, Division of Clinical Microbiology, Karolinska Institute, Stockholm, Sweden; 2 Department of Infectious Diseases, University of Gothenburg, Sahlgrenska University Hospital, Gothenburg, Sweden; 3 The Swedish Institute for Infectious Disease Control, Solna, Sweden; 4 Department of Microbiology, Tumour and Cell Biology MTC, Karolinska Institute, Stockholm, Sweden; 5 Center for Infectious Medicine, Department of Medicine, Karolinska Institute, Stockholm, Sweden; 6 Department of Mathematical Statistics, Chalmers University of Technology, Gothenburg, Sweden; 7 Department of Clinical Virology, University of Gothenburg, Sahlgrenska University Hospital, Gothenburg, Sweden; 8 Department of Infectious Diseases, Karolinska Institute, Stockholm, Sweden

## Abstract

**Background:**

The latency of HIV-1 in resting CD4^+ ^T-lymphocytes constitutes a major obstacle for the eradication of virus in patients on antiretroviral therapy (ART). As yet, no approach to reduce this viral reservoir has proven effective.

**Methods:**

Nine subjects on effective ART were included in the study and treated with high dosage intravenous immunoglobulin (IVIG) for five consecutive days. Seven of those had detectable levels of replication-competent virus in the latent reservoir and were thus possible to evaluate. Highly purified resting memory CD4^+ ^T-cells were activated and cells containing replication-competent HIV-1 were quantified. HIV-1 from plasma and activated memory CD4^+ ^T-cells were compared with single genome sequencing (SGS) of the *gag *region. T-lymphocyte activation markers and serum interleukins were measured.

**Results:**

The latent HIV-1 pool decreased with in median 68% after IVIG was added to effective ART. The reservoir decreased in five, whereas no decrease was found in two subjects with detectable virus. Plasma HIV-1 RNA ≥ 2 copies/mL was detected in five of seven subjects at baseline, but in only one at follow-up after 8–12 weeks. The decrease of the latent HIV-1 pool and the residual plasma viremia was preceded by a transitory low-level increase in plasma HIV-1 RNA and serum interleukin 7 (IL-7) levels, and followed by an expansion of T regulatory cells. The magnitude of the viral increase in plasma correlated to the size of the latent HIV-1 pool and SGS of the *gag *region showed that viral clones from plasma clustered together with virus from activated memory T-cells, pointing to the latent reservoir as the source of HIV-1 RNA in plasma.

**Conclusion:**

The findings from this uncontrolled proof-of-concept study suggest that the reservoir became accessible by IVIG treatment through activation of HIV-1 gene expression in latently-infected resting CD4^+ ^T-cells. We propose that IVIG should be further evaluated as an adjuvant to effective ART.

## Background

Although antiretroviral treatment (ART) has substantially improved the prognosis for HIV-infected patients, it does not cure the infection. Replication-competent HIV-1 persists in a stable, latent reservoir, primarily in resting CD4^+ ^T-lymphocytes [1,2]. This reservoir enables long-term persistence of the infection during otherwise effective ART. Other cellular pools and tissue reservoirs, such as the central nervous system, may also be obstacles to the eradication of HIV-1 [3].

The present study focuses on intravenous immunoglobulin (IVIG) as an adjuvant to effective ART and investigates its potential effect on the latent reservoirs. Our interest in IVIG was prompted by observing the response of an HIV-1-infected subject with Guillain-Barré Syndrome who had been treated with IVIG and ART. Apart from a viral blip during IVIG treatment, HIV-1 RNA remained undetectable several months after the cessation of ART [4]. We hypothesized that IVIG contributed to activation of HIV-1 in latently-infected cells, leading to a transient increase in plasma viral load, and followed by a decrease in infected T-lymphocytes. These events then contributed to the relatively long period of undetectable viral load after ART interruption. The present proof-of-concept study was conducted to explore this hypothesis.

## Materials and methods

### Patients

Nine patients followed at the Department of Infectious Diseases, Sahlgrenska University Hospital, Gothenburg, Sweden, with continuous ART for ≥ 2 years and plasma HIV-1 RNA levels < 50 copies/mL ≥ 1.5 years, were included in the study. Written informed consent was obtained and the study was approved by the Research Ethics Committee at the University of Gothenburg and the Medical Products Agency of Sweden. Patient characteristics are summarized in Table 1. Seven subjects had detectable levels of replication-competent virus in the latent pool and were thus possible to evaluate regarding changes of the latent reservoir. Two patients had undetectable virus both before and after intervention with IVIG (subjects 1 and 5). All subjects received 30 g IVIG per day as intravenous infusions for five consecutive days (Kiovig^®^, Baxter Healthcare Corporation, Chicago, IL, USA). ART was continued throughout the study period.

**Table 1:** Patient characteristics

**Patient (age in years, sex)**	**Antiretroviral Treatment**	**Duration (months)**		**CD4 nadir**	**CD4 baseline**
		**treatment**	**< 50 copies/mL**	**per uL (%)**	**per uL (%)**
1 (65, M)	ZDV+3TC+EFV	78	74	180 (13%)	630 (38%)
2 (53, M)	TDF+FTC+EFV	124	82	200 (21%)	550 (45%)
3 (35, M)	ABC+ZDV+3TC+LPV/r	75	38	20 (3%)	200 (19%)
4 (36, F)	ZDV+3TC+LPV/r	54	48	50 (6%)	330 (28%)
5 (58, M)	ABC+DDI+EFV	131	71	40 (4%)	240 (19%)
6 (36, M)	ZDV+3TC+EFV	36	35	120 (7%)	230 (18%)
7 (43, M)	D4T+TDF+FTC+LPV/r	35	21	40 (4%)	530 (14%)
8 (45, M)	TDF+FTC+NVP	82	72	30 (2%)	270 (19%)
9 (43, M)	TDF+FTC+AZV/r	78	67	90 (17%)	920 (42%)

3TC, lamivudine; ABC, abakavir; AZV/r, atazanavir/ritonavir; EFV, efavirenz; D4T, stavudine; DDI, didanosine; FTC, emtricitabine; LPV/r, lopinavir/ritonavir; NVP, nevirapine; TDF, tenofovir; ZDV, zidovudine

### Purification and quantification of resting memory CD4^+ ^T-cells

Quantification of HIV-1 in resting memory T-cells was performed before, and 8–12 weeks after initiation of IVIG. Resting CD4^+ ^T-cells were isolated from peripheral blood mononuclear cells (PBMC) by negative selection. PBMCs were obtained by Ficoll-Hypaque density gradient centrifugation from 180 mL of peripheral blood. The PBMCs were washed twice with PBS Buffer (pH 7.2 to 7.4) that contained 0.1% BSA and 2 mM EDTA. After the first wash, the cells were resuspended in PBS Buffer, and after the second wash in 30 mL of culture medium (RPMI+L-glut, 10% FCS and PenStrept), then transferred to a tissue culture flask and placed flat overnight at 37°C in a 5% CO_2 _incubator to remove monocytes by adherence. The following day the monocytes-depleted PBMCs were further purified using a Dynal CD4 Negative Isolation Kit (Invitrogen, Carlsbad, CA, USA). The kit contains magnetic beads and a monoclonal antibody mix directed towards CD8, CD14, CD16a,b, CD56, CDw123, Glycophorin A, and HLA Class II DR/DP. Additional monoclonal antibodies, Mouse-anti-human CD8 and CD25 (AbD Serotec, Kidlington, Oxford, England), were added to the monoclonals in the Dynal kit and incubated at 4°C for 20 min. During the incubation period, 100 uL beads/10^7 ^cells were washed in PBS Buffer. The PBMCs were washed and magnetic beads were added for another incubation period of 15 min in RT. The bead and cell mix were put into a magnetic rack and the supernatant containing purified resting CD4^+ ^T-cells was collected. The purity of the cell supernatant was checked by flow cytometry.

Detection and quantification of latently-infected resting CD4^+ ^T-cells were performed by a limiting dilution culture assay, as previously described [5]. Serial five-fold dilutions of cells (10^6^, 200,000, 40,000, 8,000, 1,600, and 320) were set up in duplicates and induced to express replication-competent HIV-1 by exposure to 0.5 (-2.0) ug/mL of the mitogen phytohemagglutinin (PHA), 10 (-100) U/mL interleukin 2 (IL-2), and allogeneic irradiated PBMCs, ratio 1:10. The day after activation, the culture supernatants were removed and fresh culture medium was added. To allow detection of virus growth, the resting CD4^+ ^T-cells were also co-cultured with CD8^+ ^depleted CD4^+ ^lymphoblasts from healthy donors. These blasts had been prepared by stimulation with 0.5 ug/mL PHA two days before being added to the cultures. On the seventh day after activation, the cells were split and a new set of blasts were added to the cultures. The cultures were then incubated at 37°C in a humidified incubator with 5% CO_2 _and were fed and split as needed. Supernatants were collected on a weekly basis and tested for the presence of HIV-1 p24 antigen with Architect i2000 HIV-1 Ag/Ab Combo Detection System (Abbott Diagnostics, Abbott Park, IL, USA).

Statistical analysis by the maximum likelihood method provided estimates of the infected cell frequencies expressed as infectious units per million (IUPM) resting CD4^+ ^T-cells [6]. Samples with undetectable growth were estimated to 0.25 IUPM cells, i.e. half the concentration of the lowest possible estimate (0.5 IUPM) of detectable growth. The estimate 0.5 IUPM is the maximum likelihood estimate when only one of the two replicates at the highest concentration reveals presence of infected cells and all other dilutions are negative

### Quantification of HIV-1 RNA in plasma

HIV-1 RNA was analyzed in cell-free plasma using a previously described [7] modified version of the Roche Amplicor Monitor Test (Version 1.5, Roche Diagnostic Systems, Hoffman-La Roche, Basel, Switzerland). In order to yield a detection limit of 2 copies/mL, 8.25 mL of plasma were ultracentrifuged at 180,000 G in 4°C for 30 min prior to quantification [7].

### Viral RNA extraction from plasma and supernatants from activated memory T-cells

A volume of 3 mL plasma from subjects 2 and 7, containing 57 and 81 copies of HIV-1 RNA, respectively, was centrifuged at 20,000 × *g *for 1 hr at 4°C. After centrifugation, the supernatant was removed and the virion pellet resuspended in 150 uL PBS (pH 7.4). The cell-free supernatants from the cultures were collected and 150 uL was used with the addition of 5 ug cRNA for extraction of HIV-1 RNA using the RNeasy Lipid Tissue Mini Kit (Qiagen, Hilden, Germany). The 24 Plus Vacuum Manifold (Qiagen) was used for fast and efficient vacuum processing of QIAGEN spin columns. Extracted viral RNA was eluted in 30 uL of MilliQ water with the addition of 1 uL RNase inhibitor (Promega, Madison, WI, USA). The entire viral RNA extraction was used for cDNA synthesis. To avoid contamination issues, the extraction and amplification of each patient's cell culture supernatant and plasma samples were carried out separately.

### Single genome sequencing (SGS)

cDNA synthesis was performed using the ThermoScript RT-PCR System (Invitrogen) with gene-specific primer 5'-TCTTTCATTTGRTGTCCTTC-3' (HXB2 nt position 2063–2044) (0.1 uM). To obtain PCR products derived from single cDNA molecules, a modified version of a previously described method was used [8]. Designed subtype B-specific primers were selected to amplify the HIV-1 p24 region of *gag*, using a nested PCR with Platinum Taq DNA Polymerase (Invitrogen). First round PCRs used forward primer 5'-CATMTAGTATGGGCAAGCAG-3' (HXB2 nt position 886–905) and reverse primer (described above). This was followed by a nested PCR, where each PCR product was subsequently used as a template, with forward primer 5'-GTCAGCCAAAATTACCCTA-3' (HXB2 nt position 1171–1189) and reverse primer 5'-GTCAGCCAAAATTACCCTA-3' (HXB2 nt position 2048–2030). To obtain PCR products for SGS, the cDNA was diluted until approximately 30% of the PCR reactions yielded DNA product [9]. Positive nested PCRs were identified by agarose gel electrophoresis, using E-Gel 96 1% agarose (Invitrogen).

After purification, sequencing was conducted using the BigDye Terminator Version 3.1 Cycle Sequencing Kit (Applied Biosystems, Foster City, CA, USA), purified through Sephadex G-50 (GE Healthcare) in a Multi-Screen-HV Plate (Millipore, Billerica, MA, USA), and detected in the ABI PRISM 3130*xl *Genetic Analyzer (Applied Biosystems). Sequences were imported and manually edited using Sequencher software (Gene Codes, Ann Arbor, MI, USA). We obtained 10 and 15 single genomes from the plasma samples of subjects 2 and 7, respectively. The supernatant samples of cell cultures generated 23 to 38 single genomes corresponding to each time point.

### Phylogenetic analysis

Sequences were aligned using BioEdit Sequence Alignment Editor (Citeline, New York, NY, USA), with reference sequences from the HIV sequence database  to exclude the possibility of contamination. Phylogenetic trees were constructed using MEGA4.0 software (Center for Evolutionary Functional Genomics, The Biodesign Institute, Tempe, AZ, USA). Bootstrap testing (500 replicates) of phylogeny was performed using neighbor-joining, implementing pairwise deletion of gaps and gamma distribution (0.5) among sites. The sequences have been submitted to GenBank [J496870-FJ497003].

### Immunological assays

Peripheral blood CD4^+ ^and CD8^+ ^T-cell counts were measured by direct immunofluorescence in a flow cytometer.

### T-cell assays, flow cytometry, and mAbs

The following mAbs were used: anti-CD3 PE-Cy7, anti-CD4 FITC, anti-CD8 PerCP, anti-CD25 PE, anti-CD38 FITC PE-Cy7, anti-CD127 Alexa647, anti-HLA-DR APC-Cy7, anti-IFNγ FITC, anti-MIP-1β PE, and anti-IL-2 APC, all from BD Biosciences (San Diego, CA, USA). TNFα Pacific Blue from eBioscience, Anti-CD3 Pacific Blue from Dako (Copenhagen, Denmark), and Aqua Live/Dead cell exclusion marker from Invitrogen were used. For each sample 7 × 10^5 ^freshly isolated PBMC were stained in a 96-well v-bottomed plate on ice for 30 min, washed three times, and resuspended in CellFix solution (BD Biosciences); all washes were done in PBS with 5% FCS. The HIV-Gag p55 peptide pool (JPT Peptide Technologies, Berlin, Germany) and the HIV-Nef peptide pool (NIH, Germantown, MD, USA) were used to study the HIV-1-specific responses, and a CMV, EBV, and Flu (CEF) control peptide pool, as well as Staphylococcal Enterotoxin B (SEB) (SIGMA-Aldrich Logistic GmbH, Schnelldorf, Germany), were added as positive controls. The PBMCs were plated at a concentration of 1 × 10^6 ^cells/well, along with peptides at a final concentration of 2 ug/mL per peptide in the pool, and incubated at 37°C for 12 hrs. As a negative control, cells were incubated with medium only to determine the background responses for each patient. For intracellular staining of cytokines, cells were stained for surface markers before permeabilization with Perm/Fix solution (BD Biosciences) at 4°C for 20 min. Cells were then washed with Perm/Wash solution and stained for intracellular IFNγ, MIP-1β, IL-2, and TNFα for 30 min, washed three times, and resuspended in CellFix solution. Multicolor flow cytometry data was acquired on a CyAn ADP instrument (Dako) [10]. Data were analyzed using FlowJo software (Tree Star, Ashland, OR, USA).

### Cytokine analysis

Plasma samples from all patients were analyzed for the presence of IL-2 and IL-7 cytokines on a Luminex 100™ System (Luminex Corp, Austin, TX, USA). The procedure is described in a protocol supplied with the IL-2 and IL-7 Human Singleplex Bead Kits (Invitrogen). Abs from the two kits were combined, and undiluted plasma samples were thoroughly mixed, centrifuged, and filtered prior to analysis.

### Statistical analysis

Wilcoxon's Signed Rank Test was used for pairwise comparisons, the Mann-Whitney U-test for comparisons between two independent groups, and Spearman's Rank Correlation Coefficient for evaluations of correlations.

## Results

**Figure 1:**
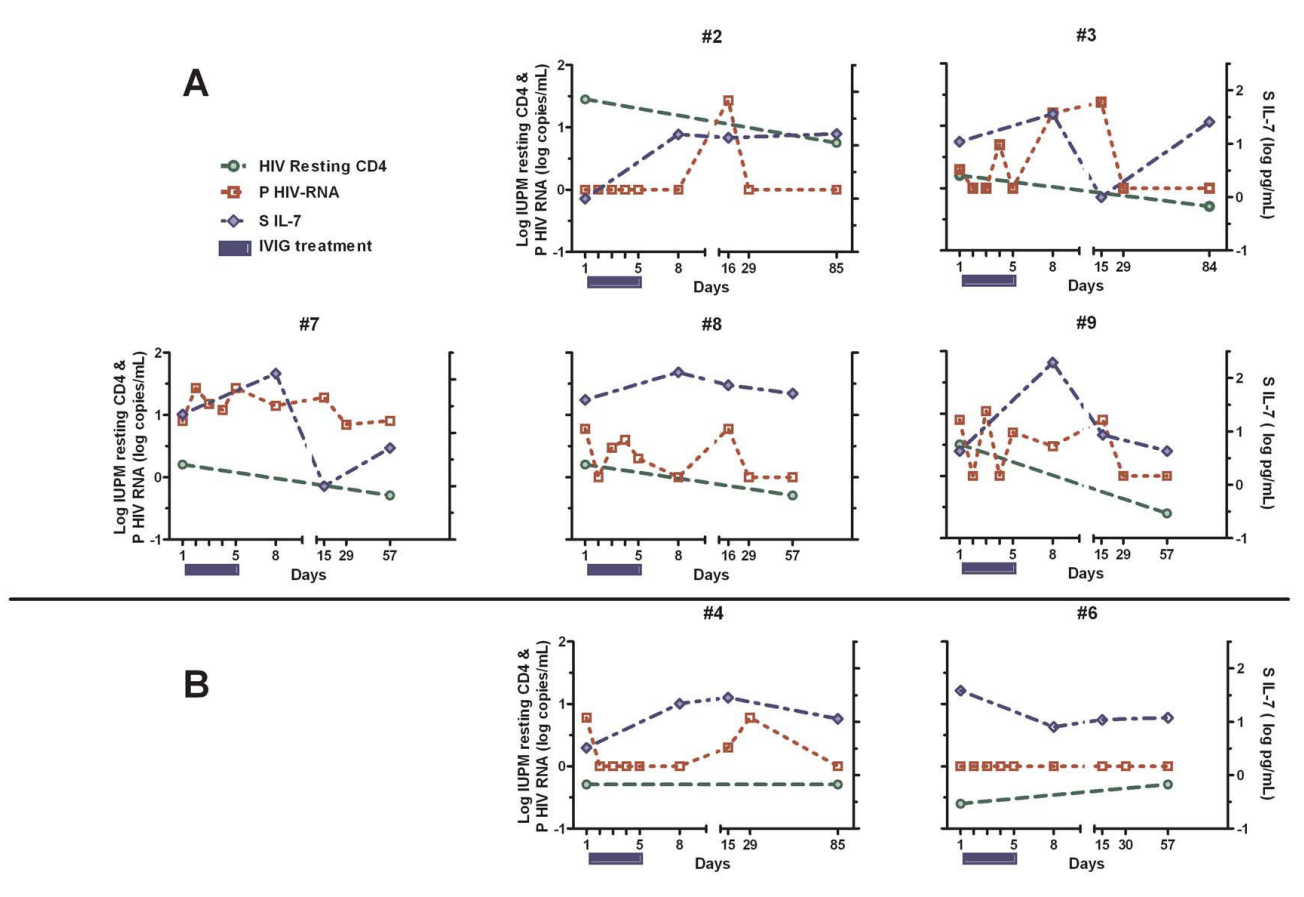
**Effect of intravenous immunoglobulin (IVIG) on resting CD4^+ ^T-cells, plasma HIV-1 RNA, and serum IL-7**. Changes in infectious units per million (IUPM) resting CD4^+ ^T-cells, plasma HIV-1 RNA, and serum interleukin-7 (IL-7) levels after addition of high-dose IVIG to continuing antiretroviral treatment. Panel A shows the five subjects with an achieved decrease of replication-competent virus in the latent reservoir. No positive effect was found in the two subjects in panel B.

**Figure 2:**
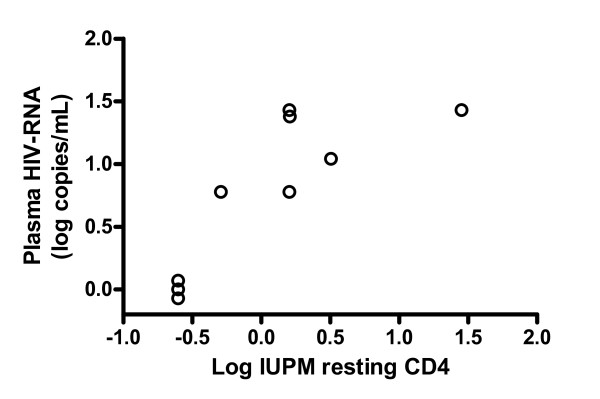
**Correlation between latently infected resting T-cells and plasma HIV-1 RNA**. Correlation between infectious units per million (IUPM) resting CD4^+ ^T-cells at baseline and maximal plasma HIV-1 RNA concentrations of the viral blip (r_s _= 0.86, *p *= 0.0045).

**Figure 3:**
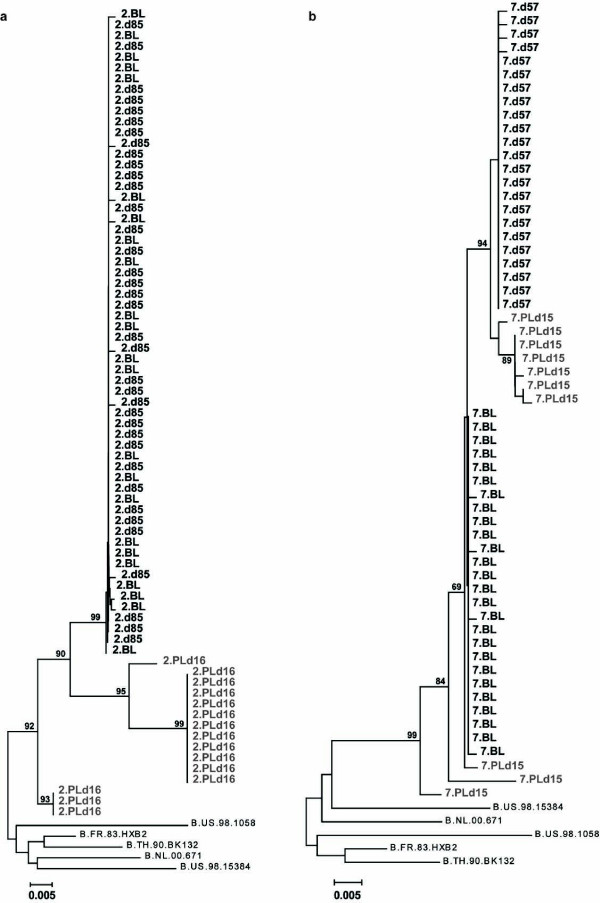
**Phylogenetic analysis of HIV-1 sequences from the latent reservoir and plasma**. Phylogenetic trees of aligned sequences obtained by SGS from patient 2 (A) and 7 (B) were determined using the neighbour-joining distance method. From patient 2, a total of 15 SGS were obtained from the plasma sample 16 days after initiation of IVIG treatment (2.PLd16); 23 SGS from the supernatant at baseline (2.BL); and 38 SGS from the supernatant of activated T-cells 85 days after initiation of IVIG treatment (2.d85). From patient 7, 10 SGS were obtained from the plasma sample 15 days after initiation of IVIG (7.PLd15); 26 SGS from the supernatant at baseline (7.BL); and 23 SGS from the supernatant 57 days after initiation of IVIG treatment (7.d57). A close relationship was found between the HIV-1 RNA from plasma-activated T-cells and the SGS from the T-cell culture. This correlation falls within the cluster of plasma sequences, implying that activation of the latent reservoir can be the source of plasma HIV-1 RNA found during IVIG treatment. Bootstrap values > 70 are indicated in the trees.

The latent HIV-1 pool decreased with a median of 68% after IVIG treatment (Table 2). When the individual subjects were scrutinized, a decrease in the latent reservoir was found in five (Figure 1a). Of the two subjects who experienced no decrease in the reservoir, one had a low pre-treatment viral load in resting cells, and in the other replication-competent virus went from undetectable to just detectable (0.5 IUPM) (Figure 1b). The five subjects with decrease of their reservoirs had a similar pattern of detectable HIV-1 RNA in plasma (6 to 27 copies/mL) two weeks after initiation of IVIG (Figure 1a). A close correlation was found between the maximal plasma viral load and levels of IUPM cells before IVIG treatment, r_s _= 0.86, *p *= 0.0045 (Figure 2). We also compared virus obtained from plasma and activated memory T-cells, using SGS of *gag *in subjects 2 and 7. Both had sufficiently high plasma viral loads to lead us to believe that sequencing would be possible. Plasma sequences were derived 15 days (subject 7) and 16 days (subject 2) after initiation of IVIG, when the plasma viral load was 19 and 27 copies/mL, respectively. In both, viruses from plasma and the T-cell reservoir were closely related, and clustered together in a distinct branch (bootstrap value > 90) in the phylogenetic trees (Figure 3). The SGS obtained from activated T-cells probably reflects an oligoclonal expansion in the culture of the most replication-competent HIV-1 in the resting T-cell population.

**Table 2:** Changes in infectious units per million (IUPM) resting CD4^+ ^T-cells after addition of intravenous immunoglobulin.

**Subject**	**IUPM**		**Decrease**
	**Baseline**	**Week 8–12**	**of pool size**
2	28.3	5.6	80%
3	1.6	0.5	68%
7	1.6	0.5	68%
8	1.6	0.5	68%
9	3.2	< 0.5	>84%

4	0.5	0.5	
6	< 0.5	0.5	

Plasma HIV-1 RNA was detectable (2–8 copies/mL) in five of the seven subjects at baseline, but in only one at follow-up after 8–12 weeks (Figure 1).

**Figure 4:**
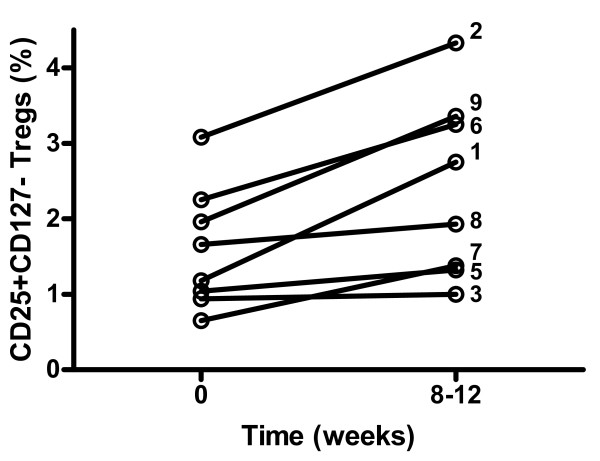
**The effect of intravenous immunoglobulin (IVIG) treatment on CD25+CD127-regulatory T-cells (Tregs)**. A consistent increase of Tregs was found after IVIG treatment, *p *= 0.0036. Patient numbers are indicated in the figure. No results were obtained from patient 4.

Serum IL-7 levels increased during the first eight days after IVIG initiation in subjects whose viral reservoirs decreased (Figure 1a). No such pattern was found for IL-2 (data not shown). We could not detect any change in CD4^+ ^T-cell counts or difference in activation of CD4^+ ^or CD8^+ ^T-cells, as measured by expression of CD25, CD38, or HLA-DR, or any effect on HIV-specific CD8^+ ^T-cell responses against Gag and Nef peptide pools (data not shown). However, a consistent increase in CD25^+^CD127^- ^regulatory T-cells (Tregs) [11], from median 1.4 (IQR: 0.96–2.2)% to 2.3 (1.3–3.3)%, was found in all subjects after IVIG treatment, *p *= 0.0036 (Figure 4).

## Discussion

The latency of HIV-1 in resting CD4^+ ^T-lymphocytes constitutes a major obstacle for the eradication of the virus in patients on ART. The decay rate of the latent reservoir in such patients is extremely slow [2,12,13] and the substantial decrease of the reservoir found in five of the subjects in this study is thus notable.

In accordance, also the plasma viremia decreased after IVIG treatment and went below the detection limit of 2 copies/mL in all but one subject. Stable low-level residual plasma viremia can normally be detected in the majority of HIV-1-infected patients on suppressive ART [14].

IVIG is currently used to treat autoimmune and inflammatory diseases. Its effects are complex and involve modulation of expression and function of Fc receptors; interference with complement activation and the cytokine network; effects on the activation and function of lymphocytes, dendritic cells, and macrophages; and provision of anti-idiotypic antibodies [15-17]. We suggest that the observed effect of IVIG on the latent reservoir may be mediated by an activation of HIV-1 gene expression in latently-infected T-cells. This hypothesis is consistent with our finding of a transient IVIG-induced increase in plasma viral load. A close correlation between the magnitude of the viral increase in plasma and the size of the latent HIV-1 pool, together with findings from SGS of the *gag *region, indicate that HIV-1 in plasma originated from the pool of latently-infected T-cells.

Expression of Fc gamma receptors on T cells is rare [18]. However, expression of the FcRgammaIIIA (CD16) can be found on small subsets of T cells and a direct effect of IVIG on such T cells can not be excluded [19,20]. However, given that most T cells lack Fc receptors, the effect of IVIG on HIV-1 activation is probably indirect and may be mediated by cytokines. It is known that IL-7 can activate virus expression, and it has, in conjunction with an anti-HIV immunotoxin, been shown to reduce the latent reservoir in a mouse model [21]. IL-7 also seems to induce proviral reactivation from resting T-lymphocytes isolated from HIV-1-infected patients on ART [22]. All subjects with decreased viral reservoirs in our study increased their serum levels of IL-7 during IVIG treatment, suggesting a role for IL-7 in mediating the effect of IVIG on the latent reservoir. The source of IL-7 detected in response to IVIG treatment is uncertain. However, in general the primary sources of IL-7 are stromal and epithelial cells, but also other sites of IL-7 production exist, including intestinal epithelium, liver and dendritic cells [23].

A consistent increase of Tregs was found after IVIG treatment. Tregs have the capacity to suppress the activation and proliferation of effector lymphocytes and thereby down-modulate chronic inflammation [24]. Expansion of Tregs by IVIG has been demonstrated previously [25]. Interestingly, HIV-1 infected individuals who control viremia without ART (so-called elite controllers) maintain high levels of these cells [26]. Tregs can limit the chronic immune activation associated with HIV-1 infection but it is unlikely that Tregs are directly involved in the activation of latent HIV-1.

Strategies to decrease the cellular reservoir of HIV-1 in latently-infected CD4^+ ^T-lymphocytes have been proposed earlier [27-32]. However, no approach has as yet proven effective. The present study suggests that IVIG may decrease the size of the latently HIV-infected memory CD4^+ ^T-cell pool. The conclusion is strengthened by the findings of transient increase in plasma virus that probably originated from resting T-cells and decreased number of subjects with detectable residual plasma viremia. However, it has to be emphasized that this was a small uncontrolled proof-of-concept study and the results need to be replicated and extended in larger studies.

## Competing interests

The authors declare that they have no competing interests.

## Authors' contributions

AL performed the purification and quantification of memory cells and the cytokine analyses under the supervision of AS. AE worked directly with patients, including sampling and the administration of drugs, and was supervised by MG. MMN was responsible for the single genome sequencing, supervised by ACK. VDG conducted the T-cell analyses; her supervisor was JKS. SN handled the statistics. BS did the two-copy HIV-1 RNA PCR. AL, AE, ACK, JKS, and AS also contributed to the design and data analyses of the study. MG originated the idea, designed the study, recruited the participants, performed data analyses, and wrote the article. All of the authors contributed to the manuscript preparation and all have seen and approved the final version.
